# Excessive daytime sleepiness and its predictors among medical and health science students of University of Gondar, Northwest Ethiopia: institution-based cross-sectional study

**DOI:** 10.1186/s12955-020-01553-3

**Published:** 2020-09-05

**Authors:** Baye Dagnew, Zewudu Andualem, Henok Dagne

**Affiliations:** 1 Department of Human Physiology, School of Medicine, College of Medicine and Health Sciences, University of Gondar, P. O. Box 196, Gondar, Ethiopia; 2 Department of Environmental and Occupational Health and Safety, Institute of Public Health, College of Medicine and Health Sciences, University of Gondar, P. O. Box 196, Gondar, Ethiopia

**Keywords:** Daytime sleepiness, Epworth sleepiness scale, Ethiopia

## Abstract

**Background:**

Excessive daytime sleepiness (EDS) is a condition of sleepiness when a person would not be expected to sleep. University students are prone to EDS due to the competitive learning environment and fragmented night sleep. No study was conducted in Ethiopia on EDS. Therefore, this study aimed to determine EDS and its predictors among University of Gondar (UoG) Medical and Health Science students.

**Methods:**

Institution-based cross-sectional study was carried out on 383 Medical and Health Science students of UoG who were recruited using a computer-generated simple random sampling technique. We used a validated Epworth daytime sleepiness tool to collect data. Epi-Info™ 7 and Stata 14 were used for data entry and analysis, respectively. Bivariable and multivariable binary logistic regression analyses were performed to find out predictors. Odds ratio with 95% uncertainty interval were computed. In the final model, a variable with a *p* < 0.05 was declared as a predictor of EDS.

**Results:**

Three hundred and eighty-three students completed the questionnaire. Males were 69.97% and the mean age of participants was 20.79 (±1.83) years. In the current study, the prevalence of EDS was 31.07% (95% UI: 26.62–35.91). The odds of getting EDS was 1.83 (AOR = 1.83, 95% UI: 1.14–2.96) and 1.84 (AOR = 1.84, 95% UI: 1.13–3.00) higher among students who reported night sleep behaviour disorders and depression, respectively.

**Conclusion:**

This study revealed that EDS is high and predicted by depression and night sleep behaviour disorders. These findings suggest the need to set preventive strategies such as counselling of students to reduce depression and night sleep behaviour disorders. Further studies particularly qualitative studies are required to find out more factors affecting EDS.

## Background

Normal sleep is essential for memory consolidation and cellular growth [1]. In normal adults, healthy sleep duration is estimated to be 7 to 9 h [2, 3]. Even though napping for less than 30 min during the day enhances learning by promoting alertness, EDS disrupts learning and the overall health condition [4]. It is a condition of sleepiness and increased falling asleep associated with tiredness and loss of mental alertness when a person would be expected to be awake [5, 6].

An EDS can exist among individuals with different morbidity such as asthma [7], renal failure [8], and gastro-oesophagal reflux disease [9] or apparently healthy people [10, 11]. As sleepiness is circumstance dependent [12], it also common in educational institutions as reported in high-school students of Korea with 15.9% of EDS [13], Malaysian Medical students (35.5%) [14], and Indian college students (45%) [15]. In Ethiopia, poor sleep quality was observed among 52.7% of university students [16], and 26% daytime sleepiness among college students [17]. University students particularly Medical and Health Science students have a huge academic load which leads to sleep deprivation and daytime sleepiness [18].

Multiple factors are associated with EDS such as nightmare and poor academic outcome [13]. The risk of EDS was higher among individuals with the difficulty of night sleep (insomnia) [19]. Contradictory reports have been sought by different researchers about the effect of sex on EDS. A study in Australia reported females are more likely to get EDS than males [20] whereas a study conducted in Japan revealed male sex is associated with EDS [21]. Besides this, a study in Japan showed no association of EDS with sex [22]. Metabolic disturbances, changes in neurotransmitter, and hormonal alterations are claimed for the genesis of EDS [23]. Long-term EDS can lead to poor health [24], reduced quality of life [23], higher risk of accidents [25], reduced productivity, and poor social dealings [26], psychological distress and poor academic performance [14].

In Ethiopia, there is rarity of studies regarding EDS among students and no study was conducted among health and medical students. Therefore, the current study aimed to determine the magnitude of EDS and identifying predictor factors among UoG Medical and Health Science students.

## Methods

### Study setting, design and period

This institution-based cross-sectional study was carried out at the University of Gondar, northwest Ethiopia. The actual data collection period was from 01-June-10 July, 2019.

### Population and eligibility criteria

All regular Medical and Health Science students of the University of Gondar available during the data collection period were included for this study. Students who were severely ill during data collection period were excluded from the study.

### Sample size determination and sampling technique

The sample size was determined using a single population proportion formula with the following assumptions; the magnitude of EDS (*P* = 50%; no previous study in the study area), 95% UI, the margin of error (d) = 5%. The minimum sample size was 384 and after adding a non-response rate of 5%, the final sample size was 404. We recruited study participants voluntarily from each department with proportional allocation. A computer-generated simple random sampling technique was used to recruit study participants. Facilitators explained the purpose of the study to participants. After obtaining consent from each participant, facilitators delivered the questionnaire to participants.

### Data collection instruments and procedure

We used a self-administered questionnaire to collect the data which comprised sociodemographic characteristics (sex, age in years, place of residence before coming to university, and pocket money), cigarette smoking, neurological conditions (depression, stress and night sleep behaviour disorder), and EDS assessment section.

Epworth daytime sleepiness scale [27] was used to collect data on EDS and it is validated in Ethiopia [28]. Night sleep behaviour disorder, depression and stress were assessed using rapid eye movement sleep behaviour disorder screening tool [29], Becks depression inventory second edition (BDI-II) [30], and perceived stress scale [31], respectively.

The BDI-II tool is validated in South Africa (but not yet in Ethiopia) [32] and is consisted of 21 items with total scores ranging from 0 to 63.

Night sleep behaviour disorder screening tool is validated elsewhere (not in Ethiopia) and is comprised of 10-items with a total score of 13 [29].

We used the perceived stress scale that is validated in Ethiopia [33], composed of 10 items each with five possible responses, and the overall value of a scale ranges from zero to 40.

Orientation was given for facilitators about the purpose of the study and ethical issues to dispatch the questionnaire, explain the purpose of the study, and receive the completed questionnaire. After obtaining written consent from each participant, respondents fill the questionnaire.

### Data quality control

We adapted the standard questionnaire for the assessment of EDS. Trained MSc Medical Physiology students were engaged for distributing, instructing students to fill the questionnaire and collecting them after filling. Trained students facilitated the data collection process explaining the purpose of the study.

### Study variables

#### Dependent variable

Excessive daytime sleepiness.

#### Independent variables

Sex of participants, place of residence before coming to university, age in years, monthly pocket money in ETB, year of study, cigarette smoking, stress, depression, the field of study, and night sleep behaviour disorder.

### Operational/term definitions

#### Excessive daytime sleepiness

A person who scored 11 and above from the total score of 24 was considered as having EDS [27].

Night sleep behaviour disorder is a sleep disorder manifested by rapid eye movement, and problematic behaviours like dream enactment, talking, sleepwalking and atonia [34]. A person is considered as having night sleep behaviour disorder when he/she scored 5 and above the screening items [35].

#### Depression

It was assessed with Beck’s depression inventory second edition (BDI-II) revised in 1996. A person with a score of 21 and above from the total scores of BDI-II was considered as depressed [36].

#### Stress

A person is defined stressed when he/she scored 5 and above of the 10 item questions of perceived stress scale (PSS-10) [37].

### Data management and statistical analysis

Data entry was performed using Epi-Info™ version 7.1. After inspection of its completeness and consistency, it was then exported into Stata 14 for statistical analysis. Categorical descriptive results were expressed using an actual number (frequency) and continuous variables were stated using mean value, range, and standard deviation. For determining measures of association between independent and dependent variables binary logistic regression was performed. The bivariable analysis was performed to determine the simple association between independent variables and EDS to select variables for multivariable analysis. Variables in bivariable analysis with a *p* < 0.2 were selected for multivariable logistic regression. In multivariable analysis, variables with a *p* < 0.05 with 95% UI were treated as predictors of EDS. Hosmer and Lemshow goodness of fit was performed to assess model fitness at *p* > 0.05. Besides *p*-values, crude odds ratio and adjusted odds ratio with 95% UI were reported.

### Reliability

We performed Cronbach’s alpha coefficient to test the reliability of the Epworth sleepiness scale which was used to assess EDS and we found a scale reliability coefficient of 0.725 which is acceptable. Furthermore, the reliability of BDI-II in this study was 0.86 (good), night sleep behaviour disorder was 0.7 (acceptable), and perceived stress scale was 0.79 (acceptable) according to Mallery P et al. [38].

## Results

### Characteristics of study participants

From a required 404 participants, 383 students took part in the study with 94.8% response rate. The mean age of the study participants was 20.79 (±1.83, range = 18–34) years. Male participants were 69.97 and 61.35% of students were second-year and above. Students with night sleep behaviour disorder, depression, and perceived stress were 46.21, 34.73, and 81.2%, respectively. Students from health science represent 78.85% of total participants and 28.2% students were from the department of Environmental and Occupational Health and Safety (Table 1).

**Table 1:** Descriptive characteristics of Medical and Health Science students of UoG, northwest Ethiopia, 2019 (*n* = 383)

Variables	Categories		Frequency	Percent (%)
Age in years	Mean(±SD) = 20.79 ± 1.83, minimum = 18 and maximum =3			
Monthly pocket money	Mean (±SD) = 689.3 ± 503.6, minimum = 50 and maximum = 3800			
Sex	Male		268	69.97
	Female		115	30.03
Residence before coming to University	Urban		224	58.49
	Rural		159	41.51
Field of study	Medicine		81	21.15
	Health sciences	Environmental and Occupational Health and Safety	108	28.2
		Health informatics	21	5.48
		Health officer	21	5.48
		Medical laboratory	36	9.40
		Nursing	28	7.31
		Pharmacy	42	10.97
		Psychiatry	33	8.62
		Physiotherapy	13	3.39
Cigarette smoking	No		359	93.73
	Yes		24	6.27
Religion	Orthodox		319	83.29
	Muslim		24	6.27
	Protestant		35	9.14
	Catholic		5	1.30
Year of study	First		148	38.64
	Second-year and above		235	61.36
Night sleep behavior disorder	No		206	53.79
	Yes		177	46.21
Depression	No		250	65.27
	Yes		133	34.73
Stress	No		72	18.80
	Yes		311	81.20
Excessive daytime sleepiness	No		264	68.93
	Yes		119	31.07

*SD* Standard deviation

### The magnitude of excessive daytime sleepiness

From the total participants, 31.07% (95% UI = 26.62, 35.91%) students reported EDS (Table 1). The mean score of Epworth sleepiness was 8.73(±4.73) (Table 2).

**Table 2:** The response of participants for each item of Epworth daytime sleepiness scale, University of Gondar, northwest Ethiopia 2019 (*n* = 383)

Items	No chance of sleeping	Slight chance of sleeping	Moderate chance of sleeping	High chance of sleeping
(Possibility of sleeping)	N (%)	N (%)	N (%)	N (%)
During sitting and reading	94(24.54)	138(36.03)	86(22.45)	65(16.97)
During watching TV	145(37.86)	127(33.16)	74(19.32)	37(9.66)
Sitting inactive in a public place (theater or meeting)	176(45.95)	104 (27.15)	66 (17.23)	37(9.66)
As a passenger in a car for an hour without break	125 (32.64)	113 (29.50)	89 (23.24)	56 (14.62)
Lying down to rest in afternoon when circumstances permit	60 (15.67)	102 (26.63)	142 (37.08)	79 (20.63)
Sitting and talking to someone	212 (55.35)	76 (19.84)	63 (16.45)	32 (8.36)
Sitting quietly after lunch without alcohol	125 (32.64)	105 (27.42)	84 (21.93)	69 (18.02)
In a car, while stopped for a few minutes in traffic	234 (61.10)	77 (20.10)	48 (12.53)	24 (6.27)
Overall Epworth sleepiness score	Mean	(±)SD	Min	max
	8.73	4.73	0	24

### Predictors of excessive daytime sleepiness

All exposure variables were tested for association using bivariable logistic regression. Age in years, cigarette smoking, year of study, and presence of night sleep behaviour disorders, depression, and stress were selected (because the *p*-value was less than 0.2) and entered into multivariable binary logistic regression. After running multivariable binary logistic regression, only night sleep behaviour disorders and depression were significantly associated with EDS. Study participants who reported night sleep behaviour disorders were 1.83 times (AOR = 1.83, 95% UI: 1.14–2.96) more likely to get EDS than those without night sleep behaviour disorders. The odds of having EDS was 1.84 (AOR = 1.84, 95% UI: 1.13–3.00) times more likely to be higher among students who reported depression than those without depression (Table 3).

**Table 3:** Predictors of excessive daytime sleepiness in bivariable and multivariable logistic regression among Medical and Health Science students of UoG, northwest Ethiopia, 2019 (*n* = 383)

Variables		Excessive daytime sleepiness		Bivariable LR	Multivariable LR
		No (%)	Yes (%)	COR (95% UI)	AOR (95% UI)
Cigarette Smoking	No	253 (70.47)	106 (29.53)	Ref	Ref
	Yes	11 (45.83)	13 (54.17)	2.82 (1.22–6.50)	1.89 (0.78–4.57)
Age in year ^c^				1.11 (0.99–1.25)	1.00 (0.88–1.15)
Year of study	1st	111 (75)	37 (25)	Ref	Ref
	≥2nd	153 (65.11)	82 (34.89)	1.61 (1.02–2.54)	1.61 (0.97–2.67)
Night sleep behavior disorders	No	159 (77.18)	47 (22.82)	Ref	Ref
	Yes	105 (59.32)	72 (40.68)	2.32 (1.49–3.61)	1.83 (1.14–2.96)^*^
Depression	No	188 (75.20)	62 (24.80)	Ref	Ref
	Yes	76 (57.14)	57 (42.86)	2.27 (1.45–3.56)	1.84 (1.13–3.00)^*^
Stress	No	55 (76.39)	17 (23.61)	Ref	Ref
	Yes	209 (67.20)	102 (32.80)	1.58 (0.87–2.86)	1.18 (0.63–2.21)

*Significant at *p* < 0.05, C = continuous variable, COR = Crude odds ratio, AOR = Adjusted odds ratio, UI = Uncertainty interval, Hosmer and Lemshow goodness of fit (Prob > chi2 = 0.4151)

## Discussion

The current study aimed to assess the magnitude of EDS and its predictors among University of Gondar Medical and Health Science students. The prevalence of EDS was 31.07% (95% UI, 26.62–35.91). Night sleep behaviour disorders and depression were predictors of EDS.

The prevalence of EDS in this study is similar to other studies from Southern University of United States (31% vs 27%) [39], Universidad San Martin de Porres in Lima, Peru (31% vs 35%) [40], Nigeria (31% vs 32.5%) [41], Southern Taiwan (31% vs 35%) [42], and Ethiopia (31% vs 26%) [17]. High magnitude of EDS in students could be explained by the highly competitive and demanding learning environment that disrupts the regular sleep time [12]. The prevalence of EDS in this study is slightly higher than other studies elsewhere as evidenced in Hunan province, central China (31% vs 24.6%) [43], Brazil (31% vs 22%) [44]. This might be because of differences in the academic culture of universities, sample size, and socioeconomic factors. Two studies from Saudi Arabia (31 vs 40%) [45] and (31 vs 68.8%) [46] reported a higher magnitude of EDS than the current study. The reason for the differences in prevalence might be attributed to variations in sample size, lifestyle, socio-cultural, academic culture, and the demographic characteristics of the study participants and difference in environmental factors.

Night sleep behaviour disorders and presence of depression were predictors of EDS among the study participants. Students who reported night sleep behaviour disorders were 1.83 times more likely to get EDS than those students without night sleep behaviour disorders. This is supported by previous studies conducted among students of the University of Saskatchewan, Canada [47], and Indian college students [45]. This association might be because night sleep behaviour disorder disturbs the quality of sleep (sleep deprivation) at night which induces EDS because of unfinished sleep periods [48, 49] and variations in melatonin hormone, vital hormone in circadian rhythm [50]. Presence of depression and night sleep disorders among university students could be due to various academic stressors [51], learning environment [52], and loneliness (homesickness) while they left family [53].

Participants who reported depression were 1.84 times more likely to have EDS than those who did not have depression. This is in agreement with another study [54]. The probable reason for this association might be a higher level of cortisol, corticotropic releasing hormone, and norepinephrine during depression lead to sleep rhythm disruption that ends up with EDS [55]. The role of cortisol on the development of depression could be explained by the neurotoxic effect of cortisol on hippocampus [56]. Other studies reported the association of EDS to stress [45, 57], Khat chewing, alcohol consumption and cigarette smoking [17], female sex [20, 22] and male sex [21]. However, these factors were not predictors of EDS in our study which might be due to variations in sample size and sociocultural factors.

The findings of this study suggest that university students are prone to EDS that is associated with depression and night sleep disturbances. If not properly handled, EDS could lead to poor academic performance [39], and psychopathology [58].

### Limitations of the study

Although the tools were widely used in Ethiopia, the tools used for the assessment of few independent variables (depression and night sleep behavior disorder) were not validated in study area. Other limitations could be social desirability and recall bias in some instances.

## Conclusion

From this study, we can conclude that EDS is high and associated with depression and night sleep behaviour disorders. This suggests the need to encourage students to get counselling on how to deal with the competitive learning environment which is important to reduce depression and night sleep behaviour disorders. Universities should establish guidance and counselling service to ease access to students. Furthermore, we recommend future researchers to undertake studies using strong designs and qualitative studies to find out the neuroendocrine connections underlying sleepiness and more factors affecting EDS.

## Data Availability

The dataset containing all the required data is found at the primary author which can be accessed with a justifiable request.

## References

[1] Carskadon MA, Dement WC (2005). Normal human sleep: an overview. Principles Pract Sleep Med.

[2] Elliot DW, Charles AC, Janet CZ, Joseph MR (1980). Timing of REM and stages 3+ 4 sleep during temporal isolation in man. Sleep..

[3] Hirshkowitz M, Whiton K, Albert SM, Alessi C, Bruni O, DonCarlos L (2015). National Sleep Foundation’s updated sleep duration recommendations. Sleep health.

[4] Dhand R, Sohal H (2006). Good sleep, bad sleep! The role of daytime naps in healthy adults. Curr Opin Pulm Med.

[5] Arand D, Bonnet M, Hurwitz T, Mitler M, Rosa R, Sangal RB (2005). The clinical use of the MSLT and MWT. Sleep..

[6] Partinen M. Epidemiology of sleep disorders. Handbook of clinical neurology: Elsevier; 2011;98:275–314. available at https://www.sciencedirect.com/science/article/pii/B9780444520067000186?via%3Dihub. 10.1016/B978-0-444-52006-7.00018-6 21056193

[7] Seneviratne U, Puvanendran K (2004). Excessive daytime sleepiness in obstructive sleep apnea: prevalence, severity, and predictors. Sleep Med.

[8] Hanly PJ, Gabor JY, Chan C, Pierratos A (2003). Daytime sleepiness in patients with CRF: impact of nocturnal hemodialysis. Am J Kidney Dis.

[9] Janson C, Gislason T, De Backer W, Plaschke P, Björnsson E, Hetta J (1995). Daytime sleepiness, snoring and gastro-oesophageal reflux amongst young adults in three European countries. J Intern Med.

[10] Young TB (2004). Epidemiology of daytime sleepiness: definitions, symptomatology, and prevalence. J Clin Psychiatry.

[11] Liu X, Uchiyama M, Kim K, Okawa M, Shibui K, Kudo Y (2000). Sleep loss and daytime sleepiness in the general adult population of Japan. Psychiatry Res.

[12] Kryger MH, Roth T, Dement WC. Principles and practice of sleep medicine, 6e édition. Philadelphie, É. U.: Elsevier Saunders. 2017. available at https://scholar.google.com/scholar?hl=en&as_sdt=0%2C5&q=Kryger+MH%2C+Roth+T%2C+Dement+WC.+Principles+and+practice+of+sleep+medicine%3A+Elsevier%3B+2017.&btnG=.

[13] Joo S, Shin C, Kim J, Yi H, Ahn Y, Park M (2005). Prevalence and correlates of excessive daytime sleepiness in high school students in Korea. Psychiatry Clin Neurosci.

[14] Zailinawati A, Teng C, Chung Y, Teow T, Lee P, Jagmohni K (2009). Daytime sleepiness and sleep quality among Malaysian medical students. Med J Malaysia.

[15] Kaur G, Singh A (2017). Excessive daytime sleepiness and its pattern among Indian college students. Sleep Med.

[16] Seblewengel L, Sheila VP, Yared AT, Mahlet GT, Yemane B, Bizu G, Michelle AW. "The Epidemiology of Sleep Quality, Sleep Patterns, Consumption of Caffeinated Beverages, and Khat Use among Ethiopian College Students", Sleep Disorders. 2012;2012(583510):11. 10.1155/2012/583510. 10.1155/2012/583510 PMC3581089 23710363

[17] Robinson D, Gelaye B, Tadesse MG, Williams MA, Lemma S, Berhane Y. Daytime sleepiness, circadian preference, caffeine consumption and Khat use among college students in Ethiopia. J Sleep Disorders Treat Care. 2013;3(1):1–14. available at10.4172/2325-9639.1000130https://www.ncbi.nlm.nih.gov/pmc/articles/PMC4015623/#. 10.4172/2325-9639.1000130 PMC4015623 24818170

[18] Azad MC, Fraser K, Rumana N, Abdullah AF, Shahana N, Hanly PJ (2015). Sleep disturbances among medical students: a global perspective. J Clin Sleep Med.

[19] Kao C-C, Huang C-J, Wang M-Y, Tsai P-S (2008). Insomnia: prevalence and its impact on excessive daytime sleepiness and psychological well-being in the adult Taiwanese population. Qual Life Res.

[20] Boccabella A, Malouf J (2017). How do sleep-related health problems affect functional status according to sex?. J Clin Sleep Med.

[21] Kaneita Y, Ohida T, Uchiyama M, Takemura S, Kawahara K, Yokoyama E (2005). Excessive daytime sleepiness among the Japanese general population. J Epidemiol.

[22] Fatani A, Al-Rouqi K, Al Towairky J, Ahmed AE, Al-Jahdali S, Ali Y (2015). Effect of age and gender in the prevalence of excessive daytime sleepiness among a sample of the Saudi population. J Epidemiol Glob Health.

[23] Panossian LA, Veasey SC (2012). Daytime sleepiness in obesity: mechanisms beyond obstructive sleep apnea—a review. Sleep..

[24] Martikainen K, Hasan J, Urponen H, Vuori I, Partinen M (1992). Daytime sleepiness: a risk factor in community life. Acta Neurol Scand.

[25] Galetta F, Franzoni F, Fallahi P, Tocchini L, Graci F, Gaddeo C (2010). Changes in autonomic regulation and ventricular repolarization induced by subclinical hyperthyroidism. Biomed Pharmacother.

[26] Breslau N, Roth T, Rosenthal L, Andreski P (1997). Daytime sleepiness: an epidemiological study of young adults. Am J Public Health.

[27] Johns MW (1991). A new method for measuring daytime sleepiness: the Epworth sleepiness scale. Sleep..

[28] Manzar MD, Salahuddin M, Alamri M, Albougami A, Khan MYA, Nureye D (2019). Psychometric properties of the Epworth sleepiness scale in Ethiopian university students. Health Qual Life Outcomes.

[29] Stiasny-Kolster K, Mayer G, Schäfer S, Möller JC, Heinzel-Gutenbrunner M, Oertel WH (2007). The REM sleep behavior disorder screening questionnaire—a new diagnostic instrument. Mov Disord.

[30] Beck AT, Steer RA, Brown GK (1996). Beck depression inventory-II. San Antonio.

[31] Cohen S, Kamarck T, Mermelstein R. Perceived stress scale. Measuring stress: A guide for health and social scientists. 1994;10:235–83.

[32] Grothe KB, Dutton GR, Jones GN, Bodenlos J, Ancona M, Brantley PJ (2005). Validation of the Beck depression inventory-II in a low-income African American sample of medical outpatients. Psychol Assess.

[33] Manzar MD, Salahuddin M, Peter S, Alghadir A, Anwer S, Bahammam AS (2019). Psychometric properties of the perceived stress scale in Ethiopian university students. BMC Public Health.

[34] Schenck CH, Mahowald MW. REM sleep behavior disorder: Clinical, developmental, and neuroscience perspectives 16 years after its formal identification in SLEEP. Sleep: J of Sleep and Sleep Disorders Res. 2002;25(2):120–38. 10.1093/sleep/25.2.120. 10.1093/sleep/25.2.120 11902423

[35] Miyamoto M, Miyamoto T, Suzuki K, Iwanami M, Hirata K. Screening Methods for REM Sleep Behavior Disorder. SLEEP DISORDERS. 2012:181. available at https://www.intechopen.com/search?term=Miyamoto%20M%20%20Miyamoto%20T,%20Suzuki%20K,%20Iwanami%20M,%20Hirata%20K.%20Screening%20Methods%20for%20REM%20Sleep%20Behavior%20Disorder%3A%20INTECHOPEN%3B%20201.

[36] Duko B, Erdado M, Ebrahim J (2019). Prevalence and factors associated with depression among hospital admitted patients in South Ethiopia: cross sectional study. BMC research notes.

[37] Cohen S, Kamarck T, Mermelstein R. Perceived stress scale. Measuring stress: A guide for health and social scientists. 1994;10:1–2.

[38] Mallery P, George D (2003). SPSS for windows step by step: a simple guide and reference.

[39] Gaultney JF (2010). The prevalence of sleep disorders in college students: impact on academic performance. J Am Coll Heal.

[40] Whittier A, Sanchez S, Castañeda B, Sanchez E, Gelaye B, Yanez D (2014). Eveningness chronotype, daytime sleepiness, caffeine consumption, and use of other stimulants among Peruvian university students. J Caffeine Res.

[41] James BO, Omoaregba JO, Igberase OO (2011). Prevalence and correlates of poor sleep quality among medical students at a Nigerian university. Ann Nigerian Med.

[42] Huang C-F, Yang L-Y, Wu L-M, Liu Y, Chen H-M (2014). Determinants of daytime sleepiness in first-year nursing students: a questionnaire survey. Nurse Educ Today.

[43] Shen Y, Meng F, Tan SN, Zhang Y, Anderiescu EC, Abeysekera RE (2019). Excessive daytime sleepiness in medical students of Hunan province: prevalence, correlates, and its relationship with suicidal behaviors. J Affect Disord.

[44] Rodrigues RND, Viegas CAA, Abreu e Silva AAA, Tavares P (2002). Daytime sleepiness and academic performance in medical students. Arq Neuropsiquiatr.

[45] Alsaggaf MA, Wali SO, Merdad RA, Merdad LA (2016). Sleep quantity, quality, and insomnia symptoms of medical students during clinical years: relationship with stress and academic performance. Saudi Med J.

[46] BaHammam AS, Alaseem AM, Alzakri AA, Almeneessier AS, Sharif MM (2012). The relationship between sleep and wake habits and academic performance in medical students: a cross-sectional study. BMC Med Educ.

[47] van der Spuy I, Karunanayake CP, Dosman JA, McMullin K, Zhao G, Abonyi S (2017). Determinants of excessive daytime sleepiness in two first nation communities. BMC Pulmonary Med.

[48] Mahowald MW (2000). What is causing excessive daytime sleepiness? Evaluation to distinguish sleep deprivation from sleep disorders. Postgrad Med.

[49] Moore M, Meltzer LJ (2008). The sleepy adolescent: causes and consequences of sleepiness in teens. Paediatr Respir Rev.

[50] Sack RL, Auckley D, Auger RR, Carskadon MA, Wright KP, Vitiello MV (2007). Circadian rhythm sleep disorders: part I, basic principles, shift work and jet lag disorders. Sleep..

[51] Waqas A, Khan S, Sharif W, Khalid U, Ali A (2015). Association of academic stress with sleeping difficulties in medical students of a Pakistani medical school: a cross sectional survey. PeerJ..

[52] Deb S, Banu PR, Thomas S, Vardhan RV, Rao PT, Khawaja N (2016). Depression among Indian university students and its association with perceived university academic environment, living arrangements and personal issues. Asian J Psychiatr.

[53] Dagnew B, Dagne H (2019). Year of study as predictor of loneliness among students of University of Gondar. BMC Res Notes..

[54] Rose D, Gelaye B, Sanchez S, Castañeda B, Sanchez E, Yanez ND (2015). Morningness/eveningness chronotype, poor sleep quality, and daytime sleepiness in relation to common mental disorders among Peruvian college students. Psychol Health Med.

[55] Bush B, Hudson T (2010). The role of cortisol in sleep. Nat Med J.

[56] Mühlen K, Ockenfels H (1969). Morphological alterations in the diencephalon and telencephalon following disturbances to the feedback mechanism adenohypophysis-adrenal cortex. 3. Studies on the guinea pig after administration of cortisone and hydrocortisone. Z Zellforsch Mikrosk Anat.

[57] Feng G, Chen J, Yang X (2005). Study on the status and quality of sleep-related influencing factors in medical college students. Zhonghua Liu Xing Bing Xue Za Zhi.

[58] Mume CO, Olawale KO, Osundina AF (2011). Excessive daytime sleepiness, nocturnal sleep duration and psychopathology among Nigerian university students. S Afr J Psychiatry.

